# Investigating the Lymphatic System by Dual-Color Elemental Mass Spectrometry Imaging

**DOI:** 10.1155/2017/4035721

**Published:** 2017-01-29

**Authors:** Ann-Christin Niehoff, Tim Klasen, Rebecca Schmidt, Daniel Palmes, Cornelius Faber, Uwe Karst, Rebecca Hadrian

**Affiliations:** ^1^Institute of Inorganic and Analytical Chemistry, University of Muenster, 48149 Muenster, Germany; ^2^Department of Clinical Radiology, University Hospital Muenster, 48149 Muenster, Germany; ^3^Department of General and Visceral Surgery, University Hospital Muenster, 48149 Muenster, Germany

## Abstract

Secondary lymphedema accompanied with strong restrictions in quality of life is still major side effects in cancer therapy. Therefore, dedicated diagnostic tools and further investigation of the lymphatic system are crucial to improve lymphedema therapy. In this pilot study, a method for quantitative analysis of the lymphatic system in a rat model by laser ablation (LA) with inductively coupled plasma mass spectrometry imaging (ICP-MSI) is presented. As a possible lymph marker, thulium(III)(1R,4R,7R,10R)-*α*,*α*′,*α*′′,*α*′′′-tetramethyl-1,4,7,10-tetraazacyclododecane-1,4,7,10-tetraacetate (Tm-DOTMA) is introduced and compared to the clinically used magnetic resonance imaging contrast agent gadolinium(III)2,2′,2′′-(10-((2R,3S)-1,3,4-trihydroxybutan-2-yl)-1,4,7,10-tetraazacyclododecane-1,4,7-triyl)triacetate (Gd-DO3A-butrol). Gadobutrol functioned as standard contrast media in MRI lymphangiography to detect lymphatic flow qualitatively. Thus, Tm-DOTMA was investigated as lymphatic marker to detect lymphatic flow quantitatively. Both contrast agents were successfully used to visualize the lymphatic flow in successive lymph nodes in LA-ICP-MS due to lower limits of detection compared to MRI. Furthermore, the distribution of contrast agents by multicolored imaging showed accumulation in specific areas (sectors) of the lymph nodes after application of contrast agents in different areas.

## 1. Introduction

Breast cancer is the most prevalent cancer in women worldwide and represents a quarter of all female cancer cases [1]. Axilla lymph node dissection (ALND) is slowly replaced by sentinel lymph node biopsy (SLNB) as standard procedure of breast cancer treatment. As a consequence, the risk of arm lymphedema in these patients reduced significantly [2, 3]. Nevertheless, the incidence of breast cancer is still very high (estimated new cases of breast cancer worldwide in 2012: 1.676.600), whereas the mortality rates are decreasing since 1990 [1]. Gold standard in lymphedema treatment is the complex decongestive therapy (CDT). This symptomatic therapy is represented by compression garments, manual lymph drainage, exercise, and skin care to reduce the volume of the affected extremity [4]. In long-term results this therapy is still unsatisfying because of lifelong restrictions and stigmatization [5]. Surgical reconstructions of the lymphatic system are versatile and show promising results in successfully reestablishing lymphatic drain in the resected area [6]. To visualize course and occurrence of lymphatic vessels and lymph nodes, lymphoscintigraphy is used as a gold standard to visualize lymphatic function. However, this method has limited spatial resolution and suffers from a lack of anatomical correlation [7]. To improve morphological imaging of lymphatic structures, magnetic resonance lymphography (MRL) has proven to be a suitable in vivo method especially after administration of contrast agents. Advantages of this technique are the outstanding soft tissue contrast and spatial resolution [8]. MRL can be used to identify donor vessels for transplantation and illustrate lymphatic drain before and after surgical intervention [6]. Fink et al. introduced gadobutrol with its high T1-relaxivity as contrast agent for interstitial MRL in rats and investigated the contrast kinetics in lymph nodes, kidney, liver, muscle, and blood [9]. Furthermore, the use of gadolinium based contrast agents for MRL in patients with breast cancer has previously been reported by Lu et al. [10].

However, gadolinium- (Gd-) based MRL does not provide quantitative data to evaluate the functionality of lymphatics. One possible approach for quantification by MR imaging might be the administration of thulium- (Tm-)based contrast agents and detecting the highly shifted 1H MRI resonance in the case of Tm(III)(1R,4R,7R,10R)-*α*,*α*′,*α*′′,*α*′′′-tetramethyl-1,4,7,10-tetraazacyclo-dodecane-1,4,7,10-tetraacetic acid (Tm-DOTMA). This technique has previously been employed for MRI cell tracking experiments [11] or for in vivo thermography [12, 13]. However, the detection of the shifted resonances of the Tm complex by MRI requires high Tm concentrations. The combination of laser ablation and inductively coupled plasma mass spectrometry (LA-ICP-MS) offers a quantitative ex vivo analysis with low limits of detection and high spatial resolution in the low micrometer scale [14]. The use of LA-ICP-MS for imaging of MRI contrast agents has previously been reported in different biological samples, such as human skin biopsy or tumor models in mice [15, 16].

In this study, Tm-DOTMA is introduced as possible lymph marker, in addition to Gd-DO3A-butrol, and the kinetics of both contrast agents is quantitatively analyzed by LA-ICP-MS in rat lymph nodes. Furthermore, dual-color LA-ICP-MS is used to study lymph transport in the investigated organism by using these Gd- and Tm-based contrast agents.

## 2. Materials and Methods

Five male Sprague Dawley rats weighing 200–300 g were used for contrast agent application. An amount of 0.3 mmol/kg gadobutrol (Gd-DO3A-butrol, Gadovist®, Bayer Vital GmbH, Leverkusen, Germany) was injected subcutaneously under isoflurane anesthesia (1.5–2.5%). Additionally, Tm-DOTMA (thulium 1,4,7,10-tetraazacyclododecane-1,4,7,10-tetramethyl-1,4,7,10-tetraacetic acid) was injected the same manner. The dosage of Tm-DOTMA (Macrocyclics, Dallas, TX, USA, dissolved in isotonic sodium chloride with a final concentration of 200 mM), was 0.08 mmol/kg.

## 3. Results and Discussion

### 3.1. Tm-Based Contrast Agents as Lymph Marker

**Figure 1:**
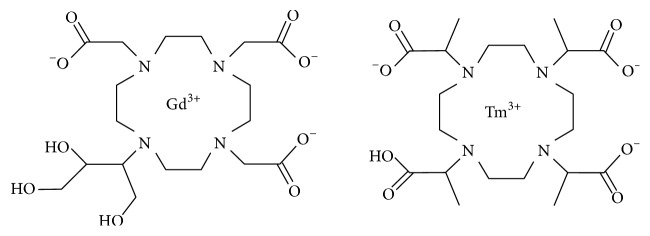
Chemical structure of the two cyclic contrast agents gadolinium(III)2,2′,2′′-(10-((2R,3S)-1,3,4-trihydroxy-butan-2-yl)-1,4,7,10-tetraazacyclododecane-1,4,7-triyl) triacetate (Gd-DO3A-butrol) and thulium(III)(1R,4R,7R,10R)-*α*,*α*′,*α*′′,*α*′′′-tetramethyl-1,4,7,10-tetraazacyclododecane-1,4,7,10-tetraacetate (Tm-DOTMA).

To investigate Tm-based contrast agents as possible lymph markers, Tm-DOTMA was administered subsequently to Gd-DO3A-butrol into the left hind paw of a rat (chemical structures of both contrast agents are shown in Figure 1). Cryosections of lymph nodes and lymph vessels were analyzed by LA-ICP-MS to verify the interstitial lymph transport.

**Figure 2:**
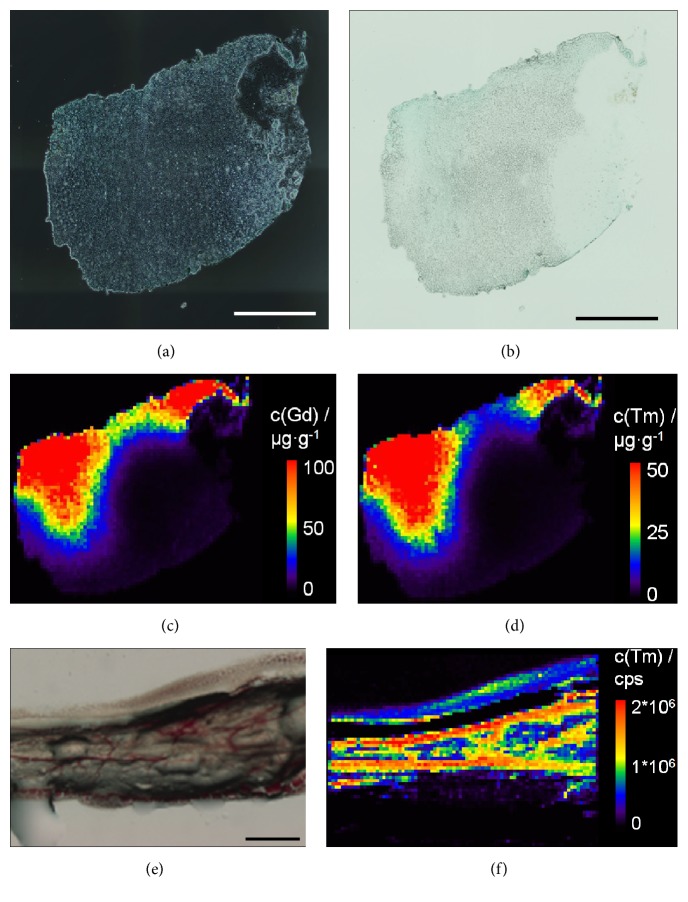
Tm-DOTMA as contrast agent for the investigation of lymphatic systems. Microscopic (a) phase contrast and (b) bright field images of a the left iliac lymph node after administration of Gd-DO3A-butrol and Tm-DOTMA in the left hind paw of a rat with corresponding LA-ICP-MS (c) Gd distribution and (d) Tm distribution. (e) Microscopic image of a lymph vessel with (f) corresponding LA-ICP-MS Tm distribution after administration of Tm-DOTMA in the left hind paw. Scale bar represents 500 *µ*m.

Figure 2 shows the iliac lymph node with corresponding Gd distribution (Figure 2(c)) and Tm distribution (Figure 2(d)). Both measurements show nearly the same distribution for Gd (max. 194 *µ*g/g) and Tm (max. 107 *µ*g/g). The lower concentration of Tm correlates with the administered concentration of 0.08 mmol/kg body weight for Tm-DOTMA and 0.3 mmol/kg body weight for Gd-DO3A-butrol. To examine lymph vessel transport of Tm-DOTMA, Tm distribution within the lymph vessel between the iliac and renal lymph node was measured (Figure 2(f)). Signal intensities up to 2*∗*10^6^ cps were detected in the lymph vessel after administration of Tm-DOTMA subcutaneously in the rat's hind paw.

**Figure 3:**
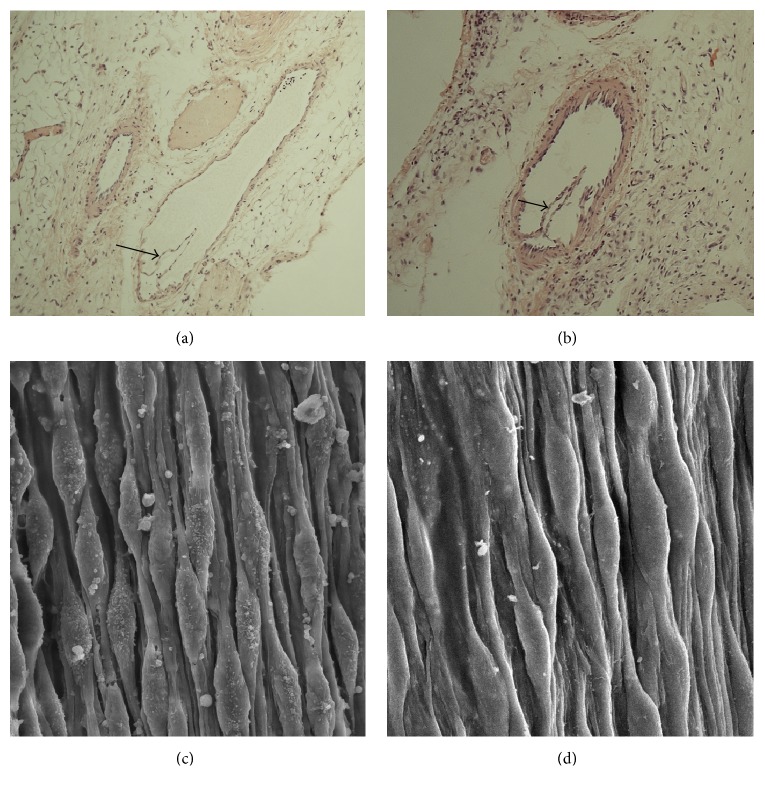
HE-staining of lymphatic vessels containing lymphatic valves revealed integrity after (a) Gd-DO3A-butrol application and (b) Gd-DO3A-butrol application and Tm-DOTMA application. Arrows indicate lymphatic valves. SEM of lymphatic endothelia was performed after application of (c) Gd-DO3A-butrol and (d) Tm-DOTMA (2200x magnification).

To investigate a possible endothelial damage caused by Tm-DOTMA administration, HE-stained lymphatic vessels were sectioned after administration of Gd-DO3A-butrol (Figure 3(a)) and Tm-DOTMA (Figure 3(b)). Histological evaluation showed that lymph endothelia and lymph valves were intact in both cases. Furthermore, scanning electron microscopy (SEM) of the lymph endothelia was performed. At 2200x magnification, the laminar surface of the lymphatic endothelia was undamaged after application of either Gd-DO3A-butrol (Figure 3(c)) or Tm-DOTMA (Figure 3(d)).

### 3.2. Kinetics of Contrast Agents in Lymph Nodes

**Figure 4:**
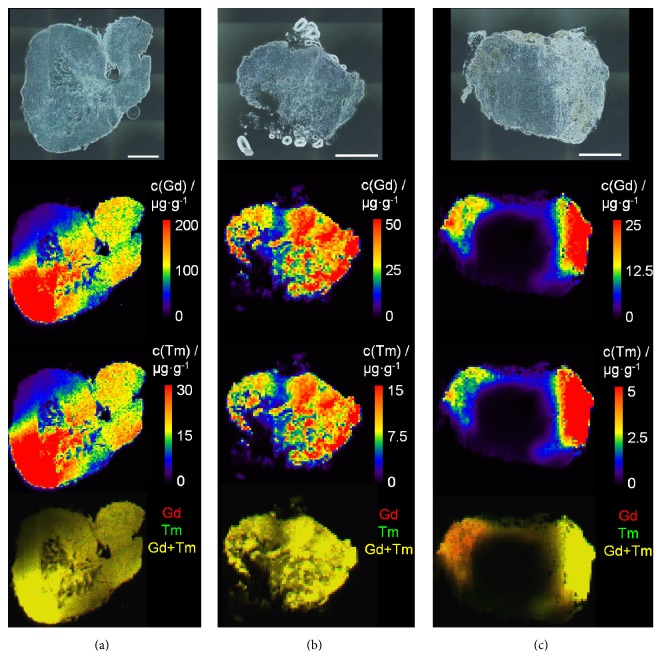
Investigation of Gd and Tm distribution in successive lymph nodes after administration of Gd-DO3A-butrol and Tm-DOTMA in the left hind paw. Microscopic images (top), Gd distribution (top-middle), Tm distribution (middle-bottom), and corresponding overlay (bottom) of lymph nodes: (a) left popliteal, (b) left iliac, and (c) left renal lymph nodes are shown. Scale bars represent 500 *µ*m.

Distribution and kinetics of Gd and Tm in subsequent lymph nodes were investigated (Figure 4). For this purpose, Tm-DOTMA and Gd-DO3A-butrol were injected subcutaneously into the left hind paw of the rat. Afterwards, the left popliteal (Figure 4(a)), left iliac (Figure 4(b)), and left renal (Figure 4(c)) lymph nodes were analyzed. From popliteal to renal lymph nodes, decreasing signal intensities for both analytes Gd and Tm were obtained. Both contrast agents showed a similar distribution in all lymph nodes within the same area of application. Maximal concentrations (calculated from 20 pixels with highest signal intensity) up to 464 *µ*g/g Gd and 83 *µ*g/g Tm for the left popliteal, 72 *µ*g/g Gd and 20 *µ*g/g Tm for the left iliac, and 40 *µ*g/g Gd and 15 *µ*g/g Tm for the left renal lymph node were detected. Lower concentrations of Tm compared to Gd are due to lower administered Tm concentrations (0.08 mmol/kg body weight for Tm-DOTMA and 0.3 mmol/kg body weight for Gd-DO3A-butrol).

Gd and Tm contrast agents showed similar but inhomogeneous distribution in all lymph nodes (Figure 4). These results can be explained by the mechanism of lymphatic drainage, as previously reported by Tammela and Alitalo [17]. The subcutaneously injected contrast agent is transported with the lymph through interendothelial gaps into the lymphatic capillaries. Lymph enters precollectors and lymphatic collectors unidirectional by negative pressure of lymphatic valves and contraction of surrounding smooth muscle cells. These afferent lymph vessels are connected to the lymph node and the lymph is transported to the subcapsular sinus through medullary sinus, to the lymph node hilus. Here, efferent lymphatic vessels spread out, join at the thoracic duct, and join the subclavian vein in the left venous angle [17]. The proposed anatomic lymph gradient from subcapsular sinus to lymph node hilus correlates with the concentration gradient presented in Figure 4.

### 3.3. Sectors in Lymph Nodes

**Figure 5:**
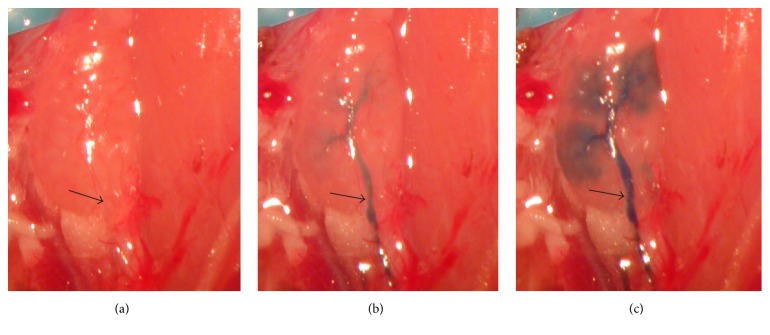
In vivo microscopic images of stained lymph nodes obtained after different periods of time. Patent blue dye was subcutaneously administered in the left hind limb of the rat in preliminary tests. The afferent lymphatic vessel is marked with an arrow. Left iliac lymph node (a) before staining, (b) after 2 min, and (c) after 5 min.

Previously, Hama et al. reported fluorescence lymphangiography of lymph nodes after administration of two fluorescence marker in different areas of a rat. Different sectors in lymph nodes were visible for the respective fluorescence marker with relatively homogenous distribution in the specific lymph node sector [18]. Our first in vivo tests after subcutaneous administration of patent blue in the hind paw of rats support these results as shown in Figure 5. After 2 minutes, the afferent lymphatic vessel is already stained with the dye and 5 minutes after injection, patent blue spreads out into different sectors in the lymph node.

**Figure 6:**
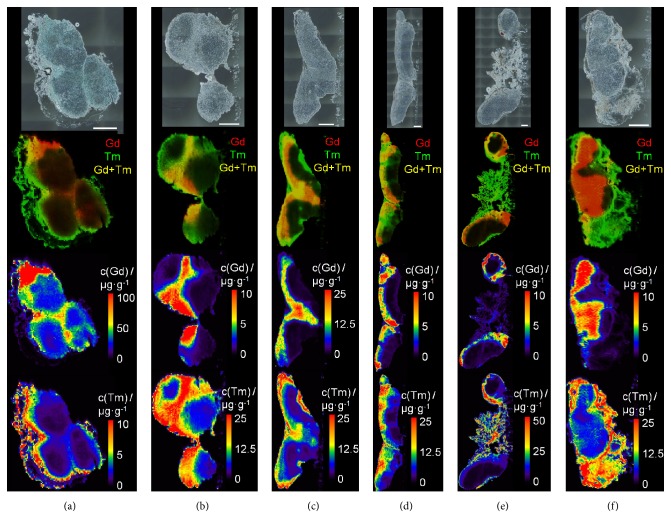
Investigation of Gd and Tm distribution after administration of Gd-DO3A-butrol in the left hind paw and Tm-DOTMA in the right forelimb. Microscopic image (top), overlay of Gd and Tm distribution (top-middle), Gd distribution (bottom-middle), and Tm distribution (bottom) of rat lymph nodes: (a) popliteal left, (b) popliteal right, (c) iliac right, (d) axillar left, (e) cranial axillar right, and (f) caudal axillar right lymph node section. Scale bar represents 500 *µ*m.

To further investigate the distribution of different contrast agents along different sectors in the lymph nodes as proposed by Hama et al. [19], two contrast agents (Gd-DO3A-butrol and Tm-DOTMA) were administered subcutaneously in different areas (Gd-DO3A-butrol in the left hind paw and Tm-DOTMA in the right forelimb). Various lymph nodes distributed in the whole animal body were analyzed by dual-colored LA-ICP-MS.  Figure 6 shows the results for Gd and Tm distribution after administration of Gd-DO3A-butrol into the left hind limb and Tm-DOTMA into the right forelimb. Highest concentration of both contrast agents could thus be obtained near the respective application area. Here, the application area is on the different side of the animal in farthest direction. Results after administration of Gd-DO3A-butrol into the left hind limb and Tm-DOTMA into the left flank are shown in Figure S1 given in the Supporting Information in Supplementary Material available online at https://doi.org/10.1155/2017/4035721. The application area is on the same side and in similar area. So the existing drainage route is similar in both contrast agents.

Results (Figure 6) show inhomogeneous distribution of Gd (red) and Tm (green), which can be seen in the Gd and Tm overlay images. Both contrast agents showed a primary accumulation in cortex and medullary sinus. Gd stays in this area, whereas Tm diffuses in the surrounding tissue of the lymph node (Figures 6(e) and 6(f)) which might be caused by the slightly higher lipophilicity of Tm-DOTMA compared to Gd-DO3A-butrol. Higher Tm concentrations compared to Gd were detected in lymph nodes located on the right side and vice versa. Highest concentration of both contrast agents could thus be obtained near the respective application area. In the case of Gd-DO3A-butrol, the left popliteal lymph node has the highest concentration of 591 *µ*g/g Gd. For Tm-DOTMA, the highest concentration in lymph tissue could be determined in the right axillar lymph nodes with 49 *µ*g/g Tm (right caudal axillar) and 82 *µ*g/g Tm (right cranial axillar). Figures 6(e) and 6(f) were the first lymph nodes on lymphatic drainage route; thus in the right axillar lymph nodes, there was the longest period of time to diffuse in surrounding fatty tissue. We consider that there will be no problem in using Tm-complexes in vivo MR lymphangiography, because of shorter measuring.

All analyzed lymph nodes reveal that only specific areas in the lymph tissue show an accumulation of the contrast agents. Obtained LA-ICP-MS images indicate high Gd or Tm concentrations in the cortex and subcapsular sinus of the lymph node as well as low signal intensities in the center. Furthermore, by two colored LA-ICP-MS, different sectors in one lymph node could be related to a specific contrast agent concentration and thus to different areas in the body.

## 4. Conclusions

We introduced Tm-DOTMA as a possible lymph marker with high correlation to Gd-DO3A-butrol distribution in the lymphatic system. No endothelial damage was observed at concentration ranges used in our study, suggesting that Tm-DOMA is a suitable lymph marker.

The investigation of the contrast agent kinetic was performed by quantitative LA-ICP-MS. Gd-DO3A-butrol and Tm-DOTMA showed similar distributions when injected into the same area. In the case of different injection areas, the closest lymph nodes showed the highest concentration of contrast agent. Both contrast agents showed primary accumulation in the cortex and subcapsular sinus of the lymph nodes.

In conclusion, this study shows the successful quantitative analysis of contrast agents within the lymphatic system by LA-ICP-MS and evaluated the lymphatic flow by dual-color imaging. Based on these results, MRI lymphangiography with Tm-DOTMA should be performed to establish in vivo quantification of the lymphatic flow after toxicological testing. Advantage of this contrast media might be the quantification of lymphatic drain in vivo in MR lymphangiography.

## Supplementary Material

The Supplementary Material shows the detailed experimental procedure, tissue preparation and results of another application area of the contrast agents to clarify differences in transport rotes in lymph nodes.

## Abbreviations

ALND: Axilla lymph node dissection
CDT: Complex decongestive therapy
Cps: Counts per second
Gd: Gadolinium
Gd-DO3A-butrol: Gadolinium(III)2,2′,2′′-(10-((2R,3S)-1,3,4-trihydroxybutan-2yl)-1,4,7,10-tetraazacyclododecane-1,4,7-triyl)triacetate
ICP-MSI: Inductively coupled plasma mass spectrometry imaging
LA: Laser ablation
LA-ICP-MS: Laser ablation inductively coupled plasma mass spectrometry
MRL: Magnetic resonance lymphography
SLNB: Sentinel lymph node biopsy
Tm: Thulium
Tm-DOTMA: Thulium(III)(1R,4R,7R,10R)-*α*,*α*′,*α*′′,*α*′′′-tetramethyl-1,4,7,10-tetraazacyclododecane-1,4,7,10-tetraacetate.
