# Supplementary material for: Investigating the Lymphatic System by Dual-Color Elemental Mass Spectrometry Imaging
Source: Contrast Media Mol Imaging. 2017 Jan 29;2017:4035721. doi: 10.1155/2017/4035721 (PMC5612703; doi:10.1155/2017/4035721)
Supplement: Supplementary file 1 — The Supplementary Material shows the detailed experimental procedure, tissue preparation and results of another application area of the contrast agents to clarify differences in transport rotes in lymph nodes. [file 4035721.f1.docx]

**Supporting Information**

**Investigating the Lymphatic System by Dual-color Elemental Mass Spectrometry Imaging**

Ann-Christin Niehoff,^‡^ Tim Klasen,^†^Rebecca Schmidt,^†^Daniel Palmes,^§^Cornelius Faber,^†^ Uwe Karst, ^*‡^ Rebecca Hadrian^§^

^‡^ Institute of Inorganic and Analytical Chemistry, University of Münster, 48149 Münster, Germany

^†^ Department of Clinical Radiology University Hospital Münster, 48149 Münster, Germany

^§^ Department of General and Visceral Surgery, University Hospital Münster, 48149 Münster, Germany

**Experimental Section**

**Animals and administration of contrast agents**

All animal procedures were approved by the local animal care committee in North Rhine-Westphalia (LANUV), Germany (AZ: 84-02-04.2014.A373). Five male Sprague Dawley rats weighing 200-300g were used for contrast agent application. An amount of 0.3 mmol/kg gadobutrol (Gd-DO3A-butrol, Gadovist®, Bayer Vital GmbH, Leverkusen, Germany) was injected subcutaneously under isoflurane anesthesia (1.5-2.5%). Additionally, Tm-DOTMA (thulium 1,4,7,10-tetraazacyclododecane-1,4,7,10-tetramethyl-1,4,7,10-tetraacetic acid) was injected the same manner. The dosage of Tm-DOTMA (Macrocyclics, Dallas, TX, USA,dissolved in isotonic sodium chloride with a final concentration of 200 mM), was 0.08 mmol/kg.The first three rats were used to show similarities in distribution of Tm in comparison to Gd within the same application area of both contrast agents subcutaneously into the left hind paw. The fourth rat got an application of Gd in the left hind paw as in the first three cases and an application of Tm in the right forelimb. The fifth rat 5 got an application of Tm in the left flank additionally to Gd application in the left hind paw.

**Tissue Preparation**

*Laser ablation inductively coupled plasma mass spectrometry (LA-ICP-MS).* 3 h after the application of contrast agents, animals were euthanized and affected lymph nodes were dissected. Collected tissue samples were cryosectioned into9 µm thick longitudinal slices.

*H&E staining.*For Hematoxylin-Eosin-staining, paraffin-embedded tissue samples of lymph vessels and iliac lymph nodes were cut into 6 µm sections and rehydrated by xylene, decreasing strengths of alcohol (100% to 0%) and finally washed with distilled water. Staining with hematoxylin and watering followed. In the end, eosin staining and dehydrating were carried out with increasing strengths of alcohol and finally xylene.

*Scanning electron microscopy (SEM).*Samples of the lymph vesselswere flushed with PBS and glutaraldehyde and fixed in glutaraldehyde. These samples were washed with PBS and fixed in osmium tetroxide and dehydrated by increasing strength of alcohol (30% to 100%). Critical point drying succeeded and tissue samples were applied to an aluminum carrier. Afterwards,samples were blade cut under a microscope and coatedwith platinum/carbon. Images were obtained using a FESEM Hitachi S800 microscope with 10 kV.

**Preparation of gelatin standards**

For the preparation of gadolinium (Gd) and thulium (Tm) standards, to 100 mg gelatin (no. 4078; Merck, Darmstadt, Germany), 900 µl Gd (ICP standard in 2% HNO_3_, Sigma-Aldrich, Steinheim, Germany) or Tm (ICP standard in 4% HNO_3_, SCP Science, Courtaboeuf, France) solution with various concentrations between 1 mg/L and 200 mg/L was added. Standards were homogenized at 52 °C and sliced into 9 µm thin sections using a cryomicrotome (CryoStar NX70, Thermo Scientific, Bremen, Germany) operated at -23 °C. Standard sections were thaw-mounted onto microscopic slides.

Determination of the total Gd and Tm concentration of the respective gelatin standards was performed by ICP-MS. Therefore, 50 mg of gelatin standard was dissolved in 2% HNO_3_ (Suprapur, Merck, Darmstadt, Germany). Samples were diluted to final concentrations ranging from 0.05 µg/L to 2 µg/L including rhodium (Rh) as internal standard in a final concentration of 1 µg/L. As calibration solution, six standards were prepared with Gd and Tm concentrations in the range of 0.01 µg/L to 5 µg/L, and 1 µg/L Rh.

Quantification of the standards was performed with a quadrupole-based iCAP Qc ICP-MS (Thermo Fisher Scientific, Bremen, Germany) equipped with a SC-4-S autosampler (Elemental Scientific, Omaha, NE). A PFA MicroFlow nebulizer (Elemental Scientific), a cyclonic spray chamber (Thermo Fisher Scientific), a quartz injector pipe with an inner diameter of 3.5 mm, a platinum sampler and platinum skimmer was used.

Analysis was performed in kinetic energy discrimination mode (KED) with 4.2 mL/min helium as collision gas. Following ICP-MS conditions were used: rf power, 1550 W; cool gas flow, 14 L/min; auxiliary gas flow, 0.8 L/min; and nebulizer gas flow, 1.1 L/min. The isotopes ^158^Gd, ^160^Gd, ^169^Tm and ^103^Rh were monitored with a dwell time of 0.1 s each.

**LA-ICP-MS experiments**

For LA-ICP-MS analysis, a laser ablation system model LSX 213 (CETAC Technologies, Omaha, NE, USA) with a 213 nm ns-pulsed Nd:YAG(neodymium-doped yttrium aluminum garnet)laser equipped with a low volume custom-built cell (V ~ 7.5 cm^3^) was used.^1^ The laser ablation parameters were optimized regarding spot size, laser energy, scanning speed and carrier gas flow based to achieve the best signal to noise ratio in combination with high scan speed. Gelatin standards and tissue sections were ablated line by line (0 µm gap) with a laser energy of 9 J/cm^2^, 25 µm spot diameter, 50 µm/s scan speed, and 20 Hz laser shot frequency.

The generated aerosol was transported to a quadrupole based mass spectrometer (iCAP Qc, Thermo Fisher Scientific) with a gas mixture of 0.8 L/min helium passing the ablation cell and 0.4 L/min argon added after the ablation cell. A thallium (Tl) solution (0.3 µg/L diluted from ICP standard in 2% HNO_3_, Merck, Darmstadt, Germany) was introduced simultaneously to monitor the sensitivity of the instrumental setup. The ICP-MS was equipped with a PFA MicroFlow nebulizer (Elemental Scientific), a cyclonic spray chamber (Thermo Fisher Scientific), a quartz injector pipe with an inner diameter of 3.0 mm, a nickel sampler and nickel skimmer. LA-ICP-MS analyses were performed in KED mode using 4.2 mL/min helium as collision gas, rf power, 1550 W; cool gas flow, 14 L/min; auxiliary gas flow, 0.8 L/min; and nebulizer gas flow, 0.5 L/min. The isotopes ^158^Gd (0.2 s),^169^Tm (0.2 s), and ^205^Tl (0.1 s) were monitored. Using this method, low limits of detection and limits of quantification (LOD, LOQ) according to the 3- and 10 σ criterion of 0.8 and 2.6 µg Gd/kg, and 0.3 and 1.1 µg Tm/kg were achieved, respectively.Data evaluation was performed using the software ImageJ 1.48f (National Institutes of Health, Bethesda, MD, USA). For determination of maximum concentrations in lymph nodes, the average of the 20 pixels with highest signal intensity was calculated.

**Results and Discussion**


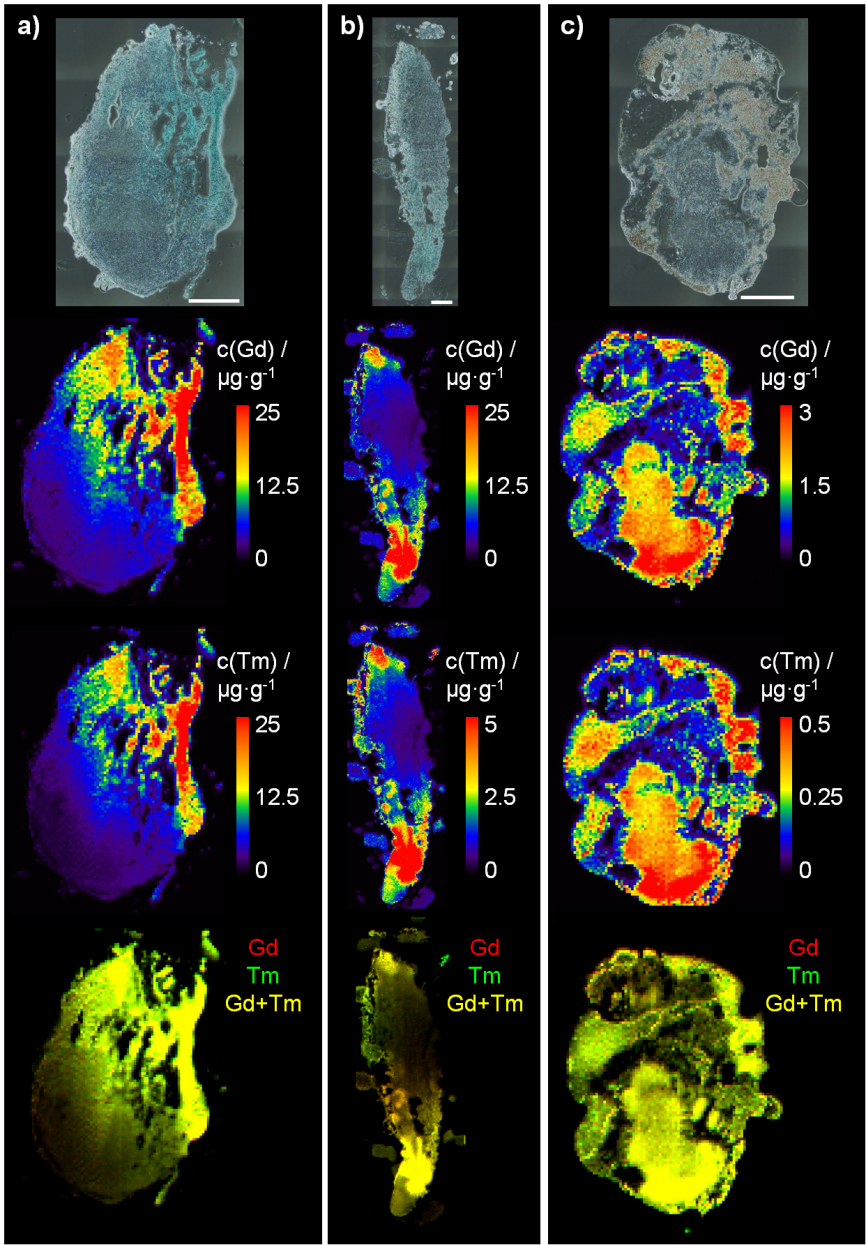


**Figure S1.** Investigation of Gd and Tm distribution after administration of Gd-DO3A-butrol in the rat´s left hind paw and Tm-DOTMA in left flank simultaneously. Microscopic image (top), Gd distribution (top-middle) and Tm distribution (bottom-middle) and corresponding overlay (bottom) of lymph nodes: (a) left popliteal lymph node, (b) left iliaclymph node left, (c) left renal lymph node.Scale bar represents 500 µm.

Figure S1 shows Gd and Tm distribution after administration of Gd-DO3A-butrol in the rat´s left hind paw and Tm-DOTMA in left flank simultaneously. The application area is on the same side and in similar area. So the existing drainage route is similar in both contrast agents Left popliteal (Figure S1a), left iliac (Figure S1b), and left renal (Figure S1c) lymph node were analyzed. Comparable results to data shown in Figure 4 were obtained concerning distribution of the contrast agent. This shows that application of two different contrast agents in similar, but not identical administration sites leads to same contrast agent distribution. Therefore, specific sectors in the lymph node are responsible for the lymph transport in a spacious area. The lymph node located near the administration site shows the highest concentration. Decreasing signal intensitieswere obtained withincreasingdistance to the administration site (popliteal 52 µg/g Gd and 47 µg/g Tm, iliac 48 µg/g Gd and 13 µg/g Tm, renal 4 µg/gGdand 0.8 µg/g Tm).

**References**

(1) Niehaus, R.; Sperling, M.; Karst, U. *J. Anal. At. Spectrom.***2015**, *30* (10), 2056.
